# Brain targeting efficiency of intranasal clozapine-loaded mixed micelles following radio labeling with Technetium-99m

**DOI:** 10.1080/10717544.2021.1951895

**Published:** 2021-07-16

**Authors:** Sinar Sayed, Fatma M. Elsharkawy, Maha M. Amin, Hesham A. Shamsel-Din, Ahmed B. Ibrahim

**Affiliations:** a Department of Pharmaceutics and Industrial Pharmacy, Faculty of Pharmacy, Cairo University, Cairo, Egypt; b Regulatory Affairs Department, Al Andalous for Pharmaceutical Industries, Giza, Egypt; c Labeled Compounds Department, Hot Labs Center, Egyptian Atomic Energy Authority, Cairo, Egypt

**Keywords:** Clozapine, polymeric nanomicellar systems, intranasal, brain targeted, radiolabeled indicator, Tetronic, Synperonic

## Abstract

The research objective is to design intranasal (IN) brain targeted CLZ-loaded polymeric nanomicellar systems (PNMS) aiming to improve central systemic CLZ bioavailability. Direct equilibrium method was used to prepare CLZ-PNMS using two hydrophobic poloxamines; Tetronic^®^ 904 (T904) and Tetronic^®^ 701 (T701) and one hydrophilic poloxamer; Synperonic^®^ PE/F127 (F127). Optimization is based on higher percent transmittance, solubilizing efficiency, and *in vitro* release after 24 h with smaller particle size was achieved using Design-Expert^®^ software. The optimized formula was further evaluated via TEM, *ex vivo* nasal permeation in addition to *in vivo* biodistribution using radiolabeling technique of the optimized formula by Technetium-99m (^99m^Tc). The optimized formula M5 has small size (217 nm) with relative high percentage of transmittance (97.72%) and high solubilization efficacy of 60.15-fold following 92.79% of CLZ released after 24 h. *Ex vivo* nasal permeation showed higher flux of 36.62 μg/cm^2^.h compared to 7.324 μg/cm^2^.h for CLZ suspension with no histological irritation. *In vivo* biodistribution results showed higher values of radioactivity percentage of the labeled optimized formula (^99m^Tc–M5) in brain and brain/blood ratio following IN administration of ^99m^Tc–M5 complex which were greater than their corresponding values following intravenous route. It is obvious that nasal delivery of CLZ-PNMS could be a promising way to improve central systemic CLZ bioavailability.

## 1. Introduction

Schizophrenia is a chronic complex psychiatric disorder, which is described as heterogeneous combination of positive symptoms such as hallucinations and delusions, also there are some negative symptoms such as lacking emotional expression, in addition to cognitive symptoms as impairment in learning, memory, attention, and executive functioning (Kahn et al., 2015; Kesby et al., 2018).

Treatment goals of schizophrenia involve relapse prevention, adaptive functioning, and symptoms targeting (Patel et al., 2014). Antipsychotic drugs have been categorized into two generations: first-generation which known as typical antipsychotics and second generation which called atypical antipsychotics, clozapine (CLZ) is considered the earliest drug of the second generation (Kahn et al., 2015). Oral atypical antipsychotics are considered the first choice for schizophrenic patients as they have advantages over typical antipsychotics such as fewer side effects regarding extrapyramidal symptoms in addition to the experienced unacceptable side effects related to traditional drugs (Willner et al., 2019).

Clozapine, a dibenzodiazepine, is atypical antipsychotic drug which has a lipophilic character, it remains the most effective second-generation antipsychotic medication, as it characterized by high affinity to dopamine ‘D4’ receptors. Also, being D2 antagonists with rapid dissociation helps in minimizing the extrapyramidal symptoms, without making any disturbance in normal dopamine neurotransmission (Babkina et al., 2018). The CLZ blocking effect of serotonin 5HT2A receptor prevents the Parkinson-like motor side effects of antipsychotics (Seeman, 2014). Its immunomodulatory action most probably due to its impact on the cytokine system, particularly within the first months of therapy by raising C-reactive protein (CRP) and proinflammatory cytokines which may be related to its therapeutic effect in the schizophrenic psychosis management in alignment with an immunological theory of disorder etiology (De Berardis et al., 2018).

Clozapine is characterized by low water solubility and high permeability, so it is classified as class II in the biopharmaceutical classification system. It has a low bioavailability (27–50%) due to first pass metabolism, with maximum plasma concentration (*T*_max_) of 1.5–2 h after oral administration. Its elimination is through extensive hepatic metabolism (Lee et al., 2021).

Different CLZ nanoformulations were performed such as intranasal (IN) nanosuspension using (+)-alpha-tocopherol polyethylene glycol 1000 succinate (TPGS) and polyvinylpyrrolidone K-30 (PVP K-30), which improved its poor pharmacokinetic parameters (Patel et al., 2021a,b). Another approach for enhancing its pharmacokinetics parameters was achieved through formulation of CLZ loaded nanostructured lipid carriers as nose to brain delivery system (Patel et al., 2021a,b).

CLZ was effectively loaded into proniosomal gel and achieved transdermal drug delivery with high entrapment efficiency and good permeation manner (Tareen et al., 2020). The encapsulation of CLZ into polycaprolactone (Łukasiewicz et al., 2019) resulted in promising nanovehicles of antipsychotic drugs.

The mixed micelles of CLZ, Pluronics, and bile salt sodium deoxycholate formed stable, suitable and high drug loading delivery vehicles for hydrophobic drug CLZ (Singla et al., 2018). Another model of nanoformulations was via CLZ-loaded polymeric nanocapsules which form safe and effective delivery system for antipsychotic drugs through blood brain barrier (Łukasiewicz et al., 2017). The antipsychotic effect of CLZ increased when formulated as nanocapsules compared to free drug (Vieira et al., 2016). Clozapine and risperidone co-entrapped as PLGA nanoparticles by spray drying process which provided small particle size (PS) with fastest drug release as dual drugs delivery nanoformulation for schizophrenia treatment (Panda et al., 2014).

Intranasal drug delivery system is a substitute for oral or injectable drug delivery, as it is considered as noninvasive, cost effective, and easy method for drug application. Also, it prevents drug exposure to either first-pass clearance or peripheral circulation following oral or injectable drug delivery. Instead, the drug reaches the central nervous system directly following IN delivery resulting in high bioavailability and CNS efficiency with lower side effects (Privalova et al., 2012).

Upon formulating IN polymeric nanomicellar systems (PNMS) from a mixture of Pluronics^®^ and Tetronics^®^, polymeric mixed micelles are obtained with nanoscopic structures having the potential of regulating the drug efflux transporter (P-gp) activity, accordingly enhancing penetration of CNS drugs through BBB. P-glycoprotein is a protein expressed on capillary endothelial cells and acting as efflux transporter which prevents drug absorption and modulates BBB permeability (Dimitrijevic & Pantic, 2014). Polymers are suggested to modulate P-gp function by two main mechanisms. First, the polymers can impact mitochondrial function and energy preservation in cells producing P-gp, resulting in its function suppression. Second mechanism of P-gp activity suppression is due to the interaction of the copolymer with the P-gp containing cell membrane (Liu et al., 2014).

In this study, CLZ-loaded PNMS were formulated and evaluated following IN administration aiming to provide efficient nose-to-brain targeting delivery for schizophrenia management with high drug residence, thereby increasing central systemic CLZ bioavailability and minimizing side effects to non-targeted organs resulting from peripheral distribution. Evaluation of CLZ-PNMS was carried out through *in vitro* characterization, *ex vivo* nasal permeation, determination of nasal cytotoxicity and *in vivo* biodistribution using ^99m^Tc as radiolabeled indicator.

## 2. Experimental

### 2.1. Materials

CLZ was a kind gift from COPAD Pharma (Cairo, Egypt). Tetronic^®^ 904 and Tetronic^®^ 701 were gift samples from BASF, Germany. Synperonic^®^ PE/F-127, acetonitrile HPLC grade, and dialysis tubing cellulose membrane (molecular weight cutoff 12,000 g/mol) were procured from Sigma Aldrich (St. Louis, MO). Methanol, sodium chloride, disodium hydrogen phosphate, and potassium dihydrogen phosphate were acquired from El Nasr Pharmaceutical Company (Cairo, Egypt). Technetium-99m (^99m^Tc) was eluted as ^99m^TcO_4_^–^ from ^99^Mo/^99m^Tc generator, Radioisotope Production Facility (RPF) (Cairo, Egypt). The whole other reagents were of analytical grade.

### 2.2. Methods

#### 2.2.1. Preparation of blank and CLZ-loaded PNMS

Preparation of single and mixed blank PNMS was carried out via direct equilibrium technique (Chen et al., 2010) by first preparing a binary mixture of both Tetronic (T904 or T701) and Synperonic F127 (in different w/w % of the total polymer weight) at a concentration of 10% w/v in distilled water and the whole system was equilibrated at 37 °C for 24 h to permit polymeric micellar formation. For comparison purposes, single micellar formulae containing 10% w/v of pure T904, T701, or F127 in distilled water were also prepared referring to formulae M1, M2, and M3, respectively (Table 1).

**Table 1.** Composition of blank and CLZ-loaded PNMS.

Formula code	Polymer percent by total weight (%w/w)		
	Tetronic^®^ 904	Tetronic^®^ 701	Synperonic^®^ PE/F127
M1	100	………	………
M2	……..	100	………
M3	……..	……..	100
M4	50	……..	50
M5	20	……..	80
M6	80	………..	20
M7	……..	50	50
M8	………	20	80
M9	……..	80	20

In a shaking water bath at 37 °C, systems of single or mixed blank PNMS containing excess amount of CLZ were kept in a shaking water bath oscillating at 100 rpm for 48 h. By centrifugation (Beckman centrifuge, Fullerton, Canada), CLZ-loaded PNMS were separated at 5000 rpm for 15 min at 25 °C. Table 1 presents the composition of CLZ-loaded PNMS either single or mixed formulae.

#### 2.2.2. Characterization of blank and CLZ-loaded PNMS

##### 2.2.2.1. Cloud point (CP)

Glass vials containing single or mixed blank PNMS in a concentration of 10% w/v in distilled water were dipped in a water bath at 25 °C. Then, the temperature was gradually raised (1 °C/min) from 25 °C until there is a sharp change from clear to turbid systems. Assays were performed in duplicates (Xiuli et al., 2004) and finally, blank PNMS formulae were cooled down.

##### 2.2.2.2. Transmittance measurement (%T)

Turbidity measurement of single or mixed blank PNMS was performed by determination of %*T* using a UV-Visible 2401 PC spectrophotometer (Shimadzu Co., Kyoto, Japan) at 520 nm using distilled water as a blank. All PNMS were stabilized at room temperature before %*T* measurement (Kulthe et al., 2011; Sayed et al., 2017).

##### 2.2.2.3. Micellar solubilization of CLZ

CLZ concentration was assayed spectrophotometrically at 292 nm compared to the apparent solubility of CLZ in pure distilled water. Experiments were repeated and average of three runs (*n* = 3) were recorded. Finally, solubility factors (*f_s_*) were computed using the following equation (Salama & Shamma, 2015):

$$f_{\text{s}}=S_{\text{a}}/S_{\text{water}}$$

where *S*_a_ and *S*_water_ are CLZ apparent solubility in PNMS and in distilled water, respectively.

##### 2.2.2.4. Micellar particle size and polydispersity index (PDI)

Micellar PS and PDI of the prepared CLZ-loaded PNMS either single or mixed were determined by photon correlation spectroscopy (PCS) using a Zetasizer Nano ZS-90 instrument (Malvern Instruments, Malvern, UK) that examines the variation in light scattering due to particles Brownian movement (Sayed et al., 2017; Habib et al., 2018). Measurement was carried out for each formula by diluting 1 mL of nano-micellar dispersion with 9 mL of distilled water (10×) and placing it into a quartz cuvette at 25 ± 0.5 °C, at 90° to the incident beam using a Zetasizer Nano ZS (Malvern Instruments Ltd., Malvern, UK). All measurements were carried out in triplicates (*n* = 3).

##### 2.2.2.5. *In vitro* release study

CLZ release from the formulated single or mixed CLZ-loaded PNMS and CLZ suspension was determined using dialysis bag technique in phosphate buffer saline PBS (pH 7.4) as a release medium. Before carrying out the release experiment, an overnight soaking of cellulose dialysis bags (molecular weight cut off 12,000–14,000 Da) in the release medium was performed. Two milliliters of CLZ-loaded PNMS (each 1 mL of the optimized formula contains 1.4 mg CLZ) were first poured into the dialysis bag tied from its both ends and put into 50 mL PBS (pH 7.4) placed in a beaker and shaken using a thermostatically controlled shaker at 37 ± 0.5 °C and 100 strokes/minute, respectively. At prearranged time points (0.5, 1, 2, 3, 4, 6, 8, 12, and 24 h), samples were withdrawn for spectrophotometric assay at *λ*_max_ of 292 nm against a blank of PBS (pH 7.4). The withdrawn samples volume was compensated by adding an equal volume of fresh PBS to the release medium (Madhuri, 2014).

#### 2.2.3. Optimization of the prepared CLZ-loaded PNMS formulae

CLZ-loaded PNMS were formulated using a 2^1^.3^1^ mixed full factorial design to study the impact of different nanomicellar variables using Design-Expert^®^ software (Version 11, Stat-Ease Inc., Minneapolis, MN) as mentioned in Table 2. The independent variables were the type of Tetronic (X1) at two levels (T904 and T701), and Tetronic concentration (X2) in the total polymer weight at three levels (20, 50, and 80% w/w). The responses analyzed were: percent transmittance (%*T*), solubilization efficiency (*f*_s_), PS, % CLZ released after 6 h and 24 h (Y1, Y2, Y3, Y4, and Y5, respectively).

**Table 2.** A 2^1^.3^1^ full factorial design used to optimize the CLZ-loaded PNMS.

	Level		
	Low (–1)	Medium (0)	High (1)
Factors (independent variables)			
X1: Tetronic^®^ type	T904		T701
X2: concentration of Tetronic^®^ in the total polymer (%w/w)	20	50	80
Responses (dependent variables)		Constraints	
Y1: %*T*		Maximize	
Y2: *f*_s_		Maximize	
Y3: PS		Minimize	
Y4: % drug released after 6 h		Maximize	
Y5: % drug released after 24 h		Maximize	

Design-Expert software was used to suggest the optimized formulation based on the criteria on highest %*T* in addition to minimum PS, maximum *f*_s_, and % CLZ released after 6 and 24 h, respectively. The optimum CLZ-loaded PNMS dispersion having the maximum desirability value which is approximate to one was elected for additional investigations.

By comparing the variance in the predicted *R*^2^ and adjusted *R*^2^ value, the precision of the model can be determined. If the difference between them within range of 0.2, they considered to be in a good agreement (Annadurai et al., 2008).

#### 2.2.4. *In vitro* characterization of the optimized CLZ-loaded PNMS formula

##### 2.2.4.1. Differential scanning calorimetry (DSC)

Thermal analysis of CLZ powder, Tetronic T904, Pluronic F127, optimized blank PNMS, and CLZ-loaded PNMS formula was carried out using DSC by heating 2 mg of each sample in aluminum pans at a heating rate of 10 °C/min under inert nitrogen flow 25 mL/min with a temperature range of 0–220 °C using Shimadzu differential scanning calorimeter (DSC-50, Kyoto, Japan) (Sayed et al., 2020).

##### 2.2.4.2. Transmission electron microscopy (TEM)

Test was performed by diluting the nanomicellar suspension 10 times with distilled water then one drop of the diluted optimized formula was deposited on a copper grid and negatively stained with 2% w/v phosphotungstic acid and dried at room temperature to be examined by TEM (JEOL, Tokyo, Japan) operated at an accelerating voltage of 80 kV (Ahmed et al., 2020).

##### 2.2.4.3. Physical stability

The optimized formula was subjected to stability study for 3 months by storing it in a sealed glass at room temperature and ambient conditions (25 ± 5 °C/60% RH) to allow the evaluation of physical stability of the optimized formula (Sayed et al., 2017). Percent drug content, PS, and percent CLZ released after 6 h and 24 h were measured at 0 time and after 3 months of storage. Results were compared using SPSS^®^ program (IBM SPSS statistics, virgin 22, Armonk, NY), where similarity factor (*f*_2_) was used to differentiate between the release profiles of CLZ before and after storage. Similarity factor ‘*f*_2_’ was computed by applying the given equation (Elsayed & Sayed, 2017):

$$f_{2}=50\times \log\left\{{\left[1+\left(\frac{1}{n}\right)\sum_{j=1}^{n}{\left|R_{t}-T_{t}\right|}^{2}\right]}^{-0.5}\times 100\right\}$$

where *R_t_* and *T_t_* represent the % CLZ released of the optimized formula before and after storage respectively, at time *t*. If ‘*f*_2_’ value between 50 and 100 suggests the similarity between the two release profiles.

#### 2.2.5. *Ex vivo* characterization of the optimized CLZ-loaded PNMS formula

##### 2.2.5.1. Isolation of sheep nasal mucosa

The protocol for animal study was approved by Research Ethics Committee (code PI 2461), Faculty of Pharmacy, Cairo University, Giza, Egypt (REC-FOPCU). From a local slaughterhouse, we obtained the head of a 1.5-year-old sheep weighing 60 kg (Cairo, Egypt). The freshly excised nasal sheep mucosa was obtained by longitudinal cut across the nasal wall (lateral part) that resulted in full exposure of the nasal cavity without damage to the septum (Vandekerckhove et al., 2009; Nour et al., 2016). The mucosa was fully removed within 10 min following sacrifice as to maintain the excised tissue viability during the experiment. The nasal mucosal membrane was then cautiously detached, instantaneously washed and dipped in ice-cold Ringer’s solution (Du et al., 2006).

##### 2.2.5.2. Histopathological studies

Histopathology study was performed in order to determine the nasal sheep mucosa integrity and local cytotoxic effect when exposed to the optimized CLZ-loaded PNMS formula. Both portions either anterior or posterior sections of the nasal sheep mucosa were detached. Every section divided into segments and distributed into groups; all the groups exposed to equal volume of each treatment (2 mL) for 2 h. The mucosa treated with PBS pH 7.4, group 1 is considered as (negative control) (Jagtap et al., 2015) and the second group received optimized CLZ-loaded PNMS (Nour et al., 2016). Then, the mucosa segments were washed with distilled water and kept in formalin: saline solution (10:90) v/v for 24 h (Al-Saraj, 2010).

Specimens were cleared by xylene and inserted in paraffin at 56 °C in hot air oven for 24 h. Sledge microtome is used to prepare paraffin bees wax tissue units at 4 µm thickness. The achieved tissue sections were gathered on glass slides, deparaffinized, stained by hematoxylin and eosin stain for usual assessment via the light electric microscope (Leica, Cambridge, UK) (Sayed et al., 2021).

##### 2.2.5.3. *Ex vivo* permeation through nasal sheep mucosa

The *ex vivo* permeation studies of optimized CLZ-loaded PNMS formula and pure CLZ suspension were carried out on nasal sheep mucosa using Franz diffusion cell. The receptor compartment was occupied by methanolic phosphate buffer saline pH 7.4 (40:60% v/v) (Agrawal & Maheshwari, 2011; Kumar et al., 2021) and the nasal sheep mucosa (1 cm^2^) was placed in such a way that the mucosa epithelial surface exposed to the donor compartment and its other side faced the receptor compartment. One milliliter of the optimized formula (equivalent to 1.4 mg CLZ) was located also in the donor compartment and wrapped with paraffin film to afford occlusive conditions. A magnet was used in the receptor part to allow receiver fluid stirring and the whole assembly was preserved at 37 ± 0.5 °C. At predetermined time intervals (1, 2, 3, 4, 6, 8, 10, 12, and 24 h), samples (1 mL) were withdrawn and the amount of CLZ permeated was measured by HPLC method (Shafaat et al., 2013).

The HPLC system consisted of Quaternary Pump (Agilent model number: G1311A; Santa Clara, CA) connected to UV detector (Agilent model number: G1311A; Santa Clara, CA). The column is C18, having 250 mm long, internal diameter of 4 mm with PS of 5 μm. The chromatographic separation was performed using mobile phase consisted of 10 mM potassium dihydrogen orthophosphate (pH adjusted to 3.0 using *o*-phosphoric acid):acetonitrile in ratio 65:35, v/v and delivered at a flow rate of 1 m/min. Twenty microliters volume of injection was used. Detections were carried out at 292 nm.

The cumulative amount of CLZ permeated through the nasal sheep mucosa (μg/cm^2^) was plotted against time (h). The steady state flux or permeation rate, *J* (μg/cm^2^ h) was calculated as the amount of CLZ passing across 1 cm^2^ of the permeation membrane per unit time and computed from the slope of the curve linear section as follows (Younes et al., 2018):

$$J=\frac{\text{amount permeated}}{A.T.}$$

where *T* is the permeation time and *A* is the surface area.

Enhancement ratio was computed by dividing the *J* of the respective formulation by the *J* of the control formulation as follows (Eiler et al., 2019):

$$\mathrm{ER}=\frac{J\text{ of the formulation}}{J\text{ of CLZ suspension}}$$

#### 2.2.6. *In vivo* characterization of the optimized CLZ-loaded PNMS formula

##### 2.2.6.1. Radiolabelling using ^99m^Tc

Direct labeling technique was used for radiolabeling of the optimized CLZ-loaded PNMS formula with technetium-99 (^99m^Tc) as a tracer (Ibrahim et al., 2014; Nour et al., 2016; Sakr et al., 2017). Sodium dithionite (Na_2_S_2_O_4_) has been used as a reducing agent for direct radiolabeling of the optimum formula with ^99m^Tc (*t*_1/2_=6 h) to skip the interference of colloidal stannic oxide with the biodistribution results, when the most familiar reducing agent (stannous chloride) is used (Qi et al., 1996; Geskovski et al., 2013).

The effect of variation of reaction conditions (sodium dithionite amount, optimum formula amount, pH of medium and reaction time) on the efficiency of radiolabeling procedure was studied and optimized in order to maximize the radiolabeling yield. In penicillin vials (10 mL), 0.1–1.5 mL of optimum formula was added to various amounts (0.1–2.5 mg) of Na_2_S_2_O_4_. 0.1 mL of technetium Tc-99m pertechnetate (^99m^TcO_4_^−^, 400 MBq) freshly eluted from ^99^Mo/^99m^Tc generator and 0.1 M HCl and/or 0.1 M NaOH solutions was added for adjustment of pH of reaction mixture. The final mixture volume was adjusted to 1 mL with distilled water. That procedure was performed by shaking of reaction mixture at temperature (25 °C) for different time points (5–120 min). Furthermore, the *in vitro* stability of ^99m^Tc–formula complex was examined for determination of the most suitable duration through which the prepared complex can be used.

Each factor was examined three times and results were statistically tested using one-way ANOVA test and the level of significance was fixed at *p*< .05. During the optimization process of radiolabeling yield, two diverse chromatographic analyses (ascending paper chromatography (PC) and thin-layer chromatography (TLC)) were applied to assay and confirm the radiolabeling yield of ^99m^Tc–M5 complex. The % of ^99m^Tc–formula, free ^99m^TcO_4_^–^ and reduced hydrolyzed (^99m^TcO_2_) colloid was determined by two various mobile phases (Hall et al., 1998; Shamsel-Din & Ibrahim, 2017).

The radiolabeling yield % of ^99m^Tc–formula complex was determined as follows:

$$\%\;\text{Radiolabeling}\text{yield}=\%100-\%\text{Free}{\;}^{\text{99m}}{\text{TcO}}_{4}{}^{–}-\%\;\text{Colloid}{\;}^{\text{99m}}{\text{TcO}}_{2}$$

##### 2.2.6.2. Biodistribution studies in mice

The animal studies protocol was revised and accepted by Research Ethics Committee of Faculty of Pharmacy, Cairo University, Giza, Egypt (REC-FOPCU) (PI 2461), and the experimental procedures for biological studies were made in harmony with the guidelines set by the Animal Ethics Committee – Egyptian Atomic Energy Authority (AEC-EAEA).

Biodistribution study of ^99m^Tc–radiolabeled optimized formula complex was used for evaluation of the *in vivo* performance of the CLZ optimized-loaded PNMS in Swiss albino mice following IN and intravenous (IV) administration. The biological distribution study was performed on normal male Swiss albino mice (1.5–2 months old) and having body mass of 25–30 g. For each route of administration, three mice were utilized in each time interval. The mice were accommodated under preserved nutritional and environmental circumstances during the study period. Randomly, the mice were distributed into two groups (I and II) corresponding to IV and IN administration route, respectively (Fahmy et al., 2018). Every group composed of 18 mice, three mice for predetermined time interval (5, 15, 30, 60, 120, and 180 min post administration). A volume of ^99m^Tc–optimized CLZ-loaded PNMS formula (40 MBq) having ∼64 µg CLZ (equivalent to 2.1–2.6 mg/kg body weight) was given.

Group I was given IV ^99m^Tc–optimized formula by mice tail vein injection. Group II given IN ^99m^Tc–optimized formula into the nostrils opening of mice as illustrated by Jain et al. (2010) using a Hamilton syringe attached to polyethylene pipe and 0.1 mm internal diameter at the delivery position (El-Setouhy et al., 2016), to allow animals to breathe the solution they were gently placed from the back in a slanted position during the administration.

After weighting the mice, they are anesthetized using chloroform and sacrificed at prearranged time points. Fresh blood sample was gathered by cardiac puncture. Then, various tissues/organs containing brain were detached, rinsed twice by normal saline solution and rendered free of tissue/fluid adherence and weighed. By using a NaI (Tl) γ-ray scintillation counter, each radioactivity for tissue/organ/fluid in addition to the background radioactivity was computed. Muscles, bone, and blood were assumed to be 40, 10, and 7% of the total body mass, respectively, to detect their weigh (Motaleb et al., 2011), % ID/g which is the % of the injected dose per gram (fluid, tissue, or organ) at the prearranged time points in a group consisting of three mice were computed through the following equation (Sakr et al., 2017):

$$\%\text{ ID/g }=\frac{\text{activity}\text{of}\text{tissue}\text{or}\text{fluid}\text{or}\text{organ}\times \text{100}}{\text{total}\text{injected}\text{activity}\times \text{weight}\text{of}\text{tissue}\text{or}\text{fluid}\text{or}\text{organ}}$$

Following gamma scintigraphy counting of blood and brain after I.N. and I.V. administration, pharmacokinetic parameters of CLZ biodistribution were analyzed using Phoenix^®^ WinNonlin^®^ 6.4 (Certara, L.P., St. Louis, MO) via non-compartmental analysis (Abdelbary & Tadros, 2013; Rashed et al., 2016). By plotting the radioactivity uptake of CLZ (%ID/g) in blood and brain samples against time (min) post administration, we can easily calculate the maximum concentration of CLZ uptake (*C*_max_) and the time to reach it (*T*_max_). Also, the area-under-the-curve from 0 to 180 min (AUC_(0–180)_, %ID/g min) was recorded. CLZ brain targeting evaluation was assessed using drug targeting efficiency percentage (DTE %) (Abd-Elal et al., 2016). DTE percentages (which are the time average dividing ratio of drug between brain and blood) was estimated concurring with the following equation:

$$\mathrm{DTE}\%=[\frac{(\frac{\text{AUC brain}}{\text{AUC blood}})\text{ in }}{(\frac{\text{AUC brain}}{\text{AUC blood}})\mathrm{iv}}]\times 100$$

where AUC brain is the area under the brain CLZ concentration–time curve from 0 to 180 min and AUC blood is the area under the blood CLZ concentration–time curve from 0 to 180 min.

The mean values of three determinations (±SD) were statistically examined utilizing one-way ANOVA test through SPSS program (IBM SPSS statistics, virgin 22, Armonk, NY). The results were judged significantly different when (*p* < .05) considered.

## 3. Results and discussion

Pluronics^®^ are polyethylene oxide–polypropylene oxide–polyethylene oxide (PEO–PPO–PEO) triblock copolymers. They are available with various molecular weights and PEO/PPO ratio. One of the FDA approved Pluronic is F127 which is highly outstanding member between Pluronics, F127 can encapsulate the hydrophobic drugs within its PPO segment (Sayed et al., 2017; Patel et al., 2018).

Tetronics (poloxamines) possess X-shaped structure which consists of chains of PPO–PEO blocks linked to central ethylene diamine group, the tertiary amine central groups has a major task regarding the thermodynamic stability of Tetronics (Vyas et al., 2018).

Recently, binary mixed micelles replaced monomicellar systems, to avoid low drug loading, large PS, and low stability (Kulthe et al., 2011; Ahmed et al., 2021). HLB (hydrophilic/lipophilic balance) and CMC (the critical micelle concentration) have an important role regarding the kinetic and thermodynamic stability of the micelles. Kinetic stability illustrates polymer exchange rate, micelle disassembly, and the system behavior over time while the thermodynamic stability describes the micellar system formation till reaching equilibrium (Kulthe et al., 2011; Lee et al., 2011).

The existence of copolymers having various HLB values help in optimizing thermodynamic and kinetic stabilities of the formed PNMS. The kinetic stability depends on many factors, one of them is hydrophobic/hydrophilic ratio. The high HLB Pluronic F127 (HLB = 22) increases the kinetic stability as the presence of long PEO chains causes steric hindrance for micelle aggregation. On the other hand, thermodynamic stability has a direct link to CMC value, as the CMC decreases by increasing the polymer hydrophobic part which results in an increase in the thermodynamic stability. That is why the Tetronics (T701 and T904) with lower HLB compared to Pluronic F127 increase the thermodynamic stability of the formed mixed micelles (Owen et al., 2012; Lee et al., 2011).

### 3.1. Preparation of blank and CLZ-loaded PNMS

Nanomicelles were formulated using direct equilibrium method, as it considered to be economical and simple method which enhances the physical stability and meets the industrial acceptance requirements.

A full 2^1^.3^1^ mixed factorial design was applied and analyzed statistically using Design-Expert software in randomized order to investigate the impact of possible combinations of all factors and levels on CLZ-loaded PNMS properties (Jain et al., 2011).

### 3.2. Characterization of the prepared blank and CLZ-loaded PNMS

#### 3.2.1. Cloud point

Copolymers are characterized by instant separation upon heating into two phases. Cloud point is defined as temperature at which phase separation takes place (Xiuli et al., 2004). It aids to select the storage conditions and to expect the stability of the formulation (Piazzini et al., 2019). By raising the temperature, dehydration of hydrophilic chains (PEO) occurs which leads to micelles accumulation and stability loss of the nanomicellar system. The molecular structures of the copolymers ‘lipophilic part (PPO) and the hydrophilic parts (PEO)’ affects its CP as by increasing hydrophobicity decreases the CP while increasing the hydrophilicity increases the CP (Chaudhari & Jayashree, 2014) due to the presence of longer PEO blocks that enhance the water solubility of the copolymer chains. Hence, these explain higher temperatures of phase separation.

The clouding manners of blank single micelles are totally different from polymeric nanomicellar mixtures. The hydrophobic copolymers T701 and T904 exhibited phase separation at a significantly lower temperature than hydrophilic copolymer F127, as predicted from their lower HLB, the CP values for T904, T701, and F127 were 65, 20, and 106 °C, respectively. The values of the pure hydrophilic copolymer were often higher than those of nanomixed micelles possibly due to the presence of T904 and T701 led to the formation of a more hydrophobic system (Table 3) (Chiappetta et al., 2011; Salama & Shamma, 2015).

**Table 3.** Measured parameters for blank and CLZ-loaded PNMS.

	Blank micelles		CLZ-loaded PNMS				
Formula	CP (°C)	%*T*	*f_s_*	PS (nm)	PDI	% Q6 h	% Q24 h
M1	65	81.20 ± 0.03	33.25 ± 1.35	491 ± 0.76	0.40 ± 0.04	42.37 ± 6.31	95.52 ± 3.18
M2	Less than RT	0.25 ± 0.63	35.65 ± 4.25	1400 ± 7.30	0.50 ± 0.06	45.35 ± 3.45	97.33 ± 3.2
M3	>100	98.20 ± 0.01	21.35 ± 2.76	40.50 ± 1.34	0.30 ± 0.01	40.35 ± 4.41	93.32 ± 4.94
M4	55	97.06 ± 2.18	70.13 ± 0.32	276.10 ± 4.05	0.44 ± 0.08	30.33 ± 1.47	84.71 ± 2.31
M5	65	97.72 ± 0.08	60.15 ± 1.94	217.10 ± 9.30	0.24 ± 0.01	39.25 ± 4.48	92.78 ± 3.20
M6	78	92.02 ± 0.33	78.25 ± 6.52	374.30 ± 10.04	0.50 ± 0.06	16.19 ± 2.22	55.51 ± 2.76
M7	Less than RT	32.77 ± 9.18	55.61 ± 2.50	495.90 ± 20.34	0.20 ± 0.05	36.70 ± 2.05	67.54 ± 1.09
M8	58	70.45 ± 4.35	37.03 ± 5.60	371.30 ± 5.10	0.19 ± 0.09	40.29 ± 2.32	87.18 ± 3.6
M9	Less than RT	1.03 ± 5.85	59.09 ± 2.60	614.30 ± 17.11	0.16 ± 0.06	19.89 ± 3.45	40.94 ± 0.37

#### 3.2.2. Percent transmittance (%T)

**Figure 1.**
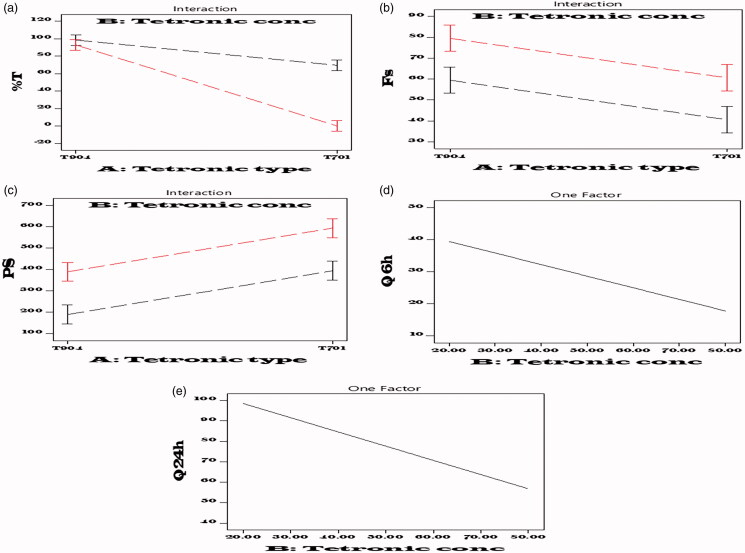
Interaction line plots of the effect of variables (Tetronic type and Tetronic conc) on the studied responses (a) Y1: %*T*, (b) Y2: Fs, (c) Y3: PS, (d) Y4: Q 6 h, and (e) Y5: Q24 h.

The effect of factors A (Tetronic type) and B (Tetronic concentration in the total polymer) and their interaction AB on the studied responses are illustrated in Figure 1(a). Turbidity measurements can demonstrate the existence of big aggregates (Kulthe et al., 2011). Also, turbidity appearance (low %*T*) in the system is usually recognized as the result of temporary detachment of huge particles or insoluble drugs (Barlow et al., 1996; Kulthe et al., 2011). A significant effect of Tetronic type and concentration was observed on %*T* values as shown in Table 4.

**Table 4.** Output data of the 2^1^×3^1^ factorial design analysis CLZ-loaded PNMS.

Responses	*R* ^2^	Adjusted *R*^2^	Predicted *R*^2^	Significant factors (*p* value)
Y1: %*T*	0.9989	0.9972	0.9846	A (0.008), B (0.0032), AB (0.0044)
Y2: *f*_s_	0.9571	0.9285	0.8443	A (0.0085), B (0.0127)
Y3: PS	0.9800	0.9666	0.8905	A (0.0025), B (0.0048)
Y4: % Q 6 h	0.9331	0.8884	0.7632	B (0.0080)
Y5: % Q24 h	0.9507	0.9178	0.8087	B (0.0057)

A is Tetronic^®^ type, B is Tetronic^®^ conc in the total polymer (%w/w), and AB is the interaction between them.

As shown in Table 3 that blank mixed micelles containing T701 has lower %*T* than the mixed micelles containing T904, this is due to the high hydrophobic character of T701 (HLB 1–7), compared to T904 (HLB 12) which possess moderate hydrophobicity, which resulted in more turbid mixed micelles. Also, by the increase in the hydrophilic part of F127, the value of %*T* increased, which suggests that the progression of aggregates from T701 and T904 has been inhibited by F127 PEO part. This is consistent with the outcomes previously announced when using hydrophobic and hydrophilic copolymers in the preparation of different nanomixed micelles (Kulthe et al., 2011; Lee et al., 2011; Abdelbary & Tadros, 2013; Salama & Shamma, 2015; Sayed et al., 2017).

#### 3.2.3. Micellar solubilization

Tetronics are X-shaped amphiphilic block copolymers containing four arms of (PEO–PPO) blocks connected to a central ethylenediamine moiety and the ability of poloxamines to form polymeric micellar systems makes them gain an essential advantage regarding the stabilization and solubilization of poorly water-soluble drugs inside their hydrophobic core (Alvarez-Lorenzo et al., 2010).

By encapsulation of CLZ within the nanomicellar systems, its apparent solubility increased up to 78 times (14.73 mg/mL) when contrasted with its aqueous solubility (0.1889 mg/mL). The PNMS of T701 and T904 revealed higher solubilization capacity than their single polymeric micelles (Table 3).

ANOVA results reveal that both factors A, B (Tetronic type and Tetronic concentration in the total polymer) had a significant effect on CLZ solubilization when encapsulated inside the polymeric nanomicelles (*p*= .0085 and .0127, respectively) (Table 4, Figure 1(b)). Regarding Tetronic type, combined T904:F127 PNMS showed higher solubilization efficacy than T701:F127 due to the presence of relatively long PEO chain which forms more stable core shell micelles which is linked to T904. In addition to the high hydrophobicity of T701 (HLB = 1–7) that results in the development of aggregated lamellar structures and its instability in aqueous environment (Kulthe et al., 2011; Salama & Shamma, 2015).

Also, regarding Tetronic concentration, upon increasing T701 and T904 ratio relative to F127, the apparent CLZ solubility was increased and this may be linked to CLZ lipophilicity which might fit more with the intermediate HLB value of T904 (12) or low HLB value of T701 (1–7) rather than the hydrophilic of F127 (HLB = 22) (Ribeiro et al., 2012; Sayed et al., 2018).

#### 3.2.4. Micellar particle size and polydispersity index

ANOVA factorial results (Table 4) showed a significant effect of both factors; A and B (Tetronic type and Tetronic concentration in the total polymer) on the size of polymeric nanomicelles (*p=* .0025 and .0048, respectively). PNMS containing (T701:F127) had higher PS (371–614 nm) than the prepared using (T904:F127) (217.1–374.3 nm) including all the ratios (Figure 1(c)) and this might be due to the more hydrophobic character of T701 entrapping more CLZ in the hydrophobic core in comparison to T904 (Rey-Rico & Cucchiarini, 2018). Also, the increase in Tetronic concentration resulted in larger PS of the prepared PNMS (Table 3) (Figure 1(c)).

Upon increasing concentration of F127, a decrease in PS from 371.3 nm to 217.1 nm of the prepared CLZ-loaded PNMS containing the highest F127% had been observed. This could be due to the formation of kinetically stable spherical micelles generated by the addition of hydrophilic copolymer that regulate and stabilize the hydrophobic interactions between T904 and T701 polypropylene oxide blocks (Salama & Shamma, 2015). The prepared micellar systems by direct equilibrium method showed smaller PS and higher drug encapsulation compared to other methods (Chen et al., 2010).

Results of PDI were used to evaluate the PS distribution (Table 3) which showed that the polydispersity value of the prepared CLZ-loaded PNMS was in the range of 0.159–0.5. It is clear that value of 0 shows greatly mono distributed systems while PDI ≥1 signifies systems with high PS distribution (Araújo et al., 2009).

#### 3.2.5. *In vitro* release study

**Figure 2.**
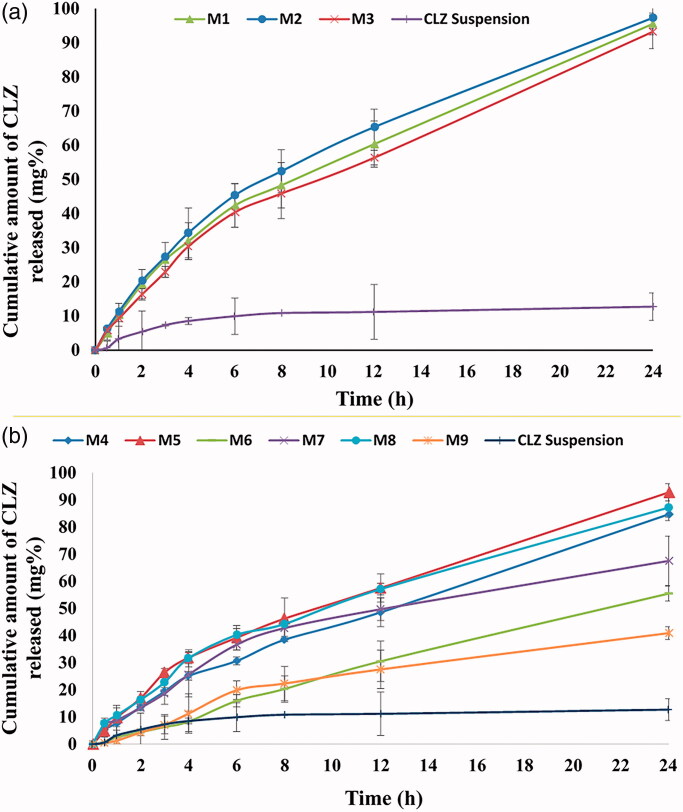
*In vitro* release profiles of CLZ-loaded single (a) and mixed CLZ-loaded PNMS (b) compared to CLZ suspension.

The profiles of CLZ release from the different prepared PNMS were performed. PNMS could sustain the release of the poorly soluble drug beside its solubilization (Nour et al., 2016). *In vitro* cumulative profiles for CLZ-loaded single and mixed PNMS release are shown in Figure 2. Single polymeric micelles have the following order of CLZ release; T701 > T904 > F127, this is due to the hydrophilic polymer's limited micellar capacity to hold the drug inside its core (Salama & Shamma, 2015).

Also, the *in vitro* release profile from CLZ PNMS was compared with CLZ suspension. The CLZ PNMS shows cumulative drug release up to 92.78% while pure CLZ suspension showed a cumulative drug release of only 12.74% at the end of 24 h that is lower than all PNMS. This may be related to the lower CLZ water solubility that resulted in its poor dissolution, as the solubility is the rate limiting step of drug dissolution (Shaik, 2015; Tambosi et al., 2018) (Figure 2(a,b)). As we mentioned before that the PNMS increased the apparent solubility of CLZ at least 78 times compared to its aqueous solubility.

ANOVA factorial results of drug release after 6 h and 24 h, showed that only Tetronic concentration had a significant effect (*p*= .0080 and .0057, respectively) (Table 4) and this means that when the concentration of Tetronic increased, the release of CLZ decreased (Figure 1(d,e)). This is might be due to the hydrophobic nature of T701 and T904 which led to more solubilization and retaining of CLZ inside their hydrophobic micelles core upon using high concentration leading to a further decrease in drug diffusion to the release medium resulting in lower *in vitro* CLZ release (Rey-Rico & Cucchiarini, 2018). In other words, the T701 and T904 hydrophobic nature makes the core environment of the PNMS more compatible with the drug which results in reduction of CLZ release rate with raise in Tetronic concentration (Batrakova et al., 2006).

### 3.3. Optimization of the prepared CLZ-loaded PNMS

Desirability was assessed using Design-Expert software for optimizing the analyzed responses by maximizing the %*T* (Y1), *f*_s_ (Y2), % released after 6 h (Y4) and 24 h (Y5) and minimizing the PS (Y3), as shown in Table 2. The responses which is only significant were taken into account. The optimized formula (M5) has the highest desirability value of 0.879, was found to be composed of T904:F127 20:80% respectively and was further evaluated.

ANOVA test was used for the evaluation of significance level of the tested factors on the previously mentioned responses in addition to their interaction, also the responses were examined separately and tailored to linear, two factor interaction (2FI) and quadratic models using linear regression (Table 4).

### 3.4. *In vitro* characterization of the optimized CLZ-loaded PNMS formula

#### 3.4.1. Differential scanning calorimetry

The DSC study was performed for CLZ powder, excipients such as (Tetronic T904 and Pluronic F127), optimized CLZ-loaded PNMS (M5) and optimized CLZ-unloaded PNMS (plain mixed micelles).

**Figure 3.**
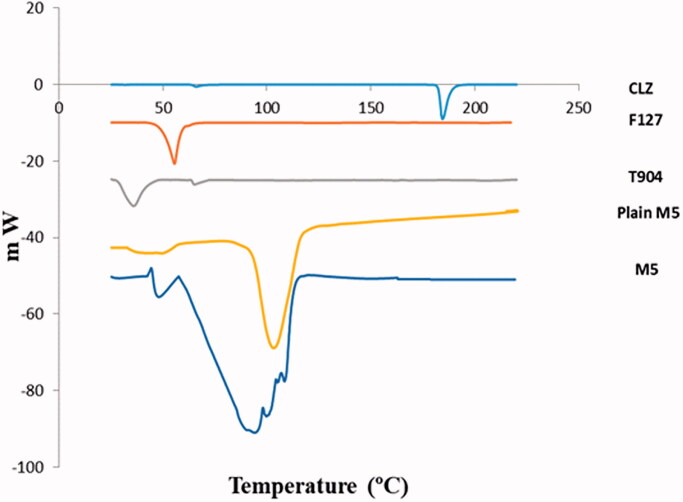
DSC thermograms of CLZ, Pluronic F127, Tetronic T904, optimized CLZ-unloaded PNMS (plain M5), and optimized CLZ-loaded PNMS (M5).

The DSC thermogram (Figure 3) shows that CLZ is characterized by sharp endothermic peak at 184.4 °C which related to its melting point which confirms its crystalline state (Abdelrahman et al., 2013). Concerning the DSC patterns of F127 and T904, endothermic peaks appeared at 56.39 °C and 32.96 °C, respectively, revealing their melting point (Kumar et al., 2011). The appearance of new thermal peaks at 100 °C related to micellization endotherm in the plain polymeric mixed micelles, also the appearance of this new peak in CLZ-loaded PNMS indicates that micellization had been occurred due to interaction of the polymers (Tetronic T904 and Pluronic F127) used in mixed micelles formation (Juggernauth et al., 2011). In addition, absence of CLZ characteristic endothermic peak could be attributed to the complete drug entrapment within the formed polymeric micelles (Leyva-Gomez et al., 2014; Sayed et al., 2017).

#### 3.4.2. Transmission electron microscopy

**Figure 4.**
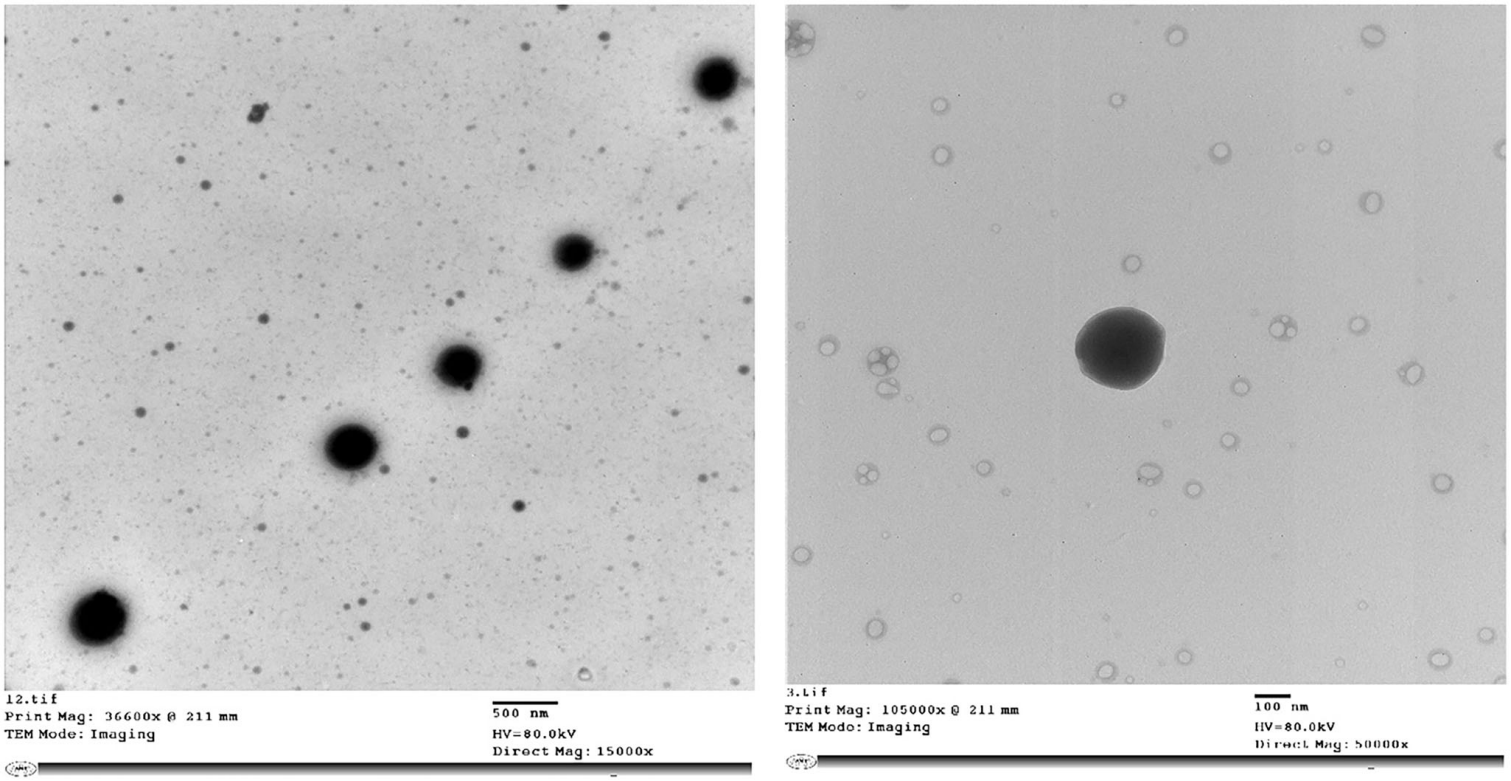
Transmission electron micrographs of the optimized CLZ-loaded PNMS at different magnifications.

TEM images of the optimized CLZ-loaded PNMS (M5) are demonstrated in Figure 4. TEM shows non-accumulated particles with spherical structure. The PS detected in TEM images was in harmony with the size measurements acquired from PS analyzer.

#### 3.4.3. Physical stability

At the end of storage period (3 months) at controlled room temperature, the optimized formula (M5), did not show any alternation in physical appearance. The recorded parameters (CLZ content %, PS, % Q6 h and % Q24 h) of the stored formula compared with the freshly prepared formula are shown in Table 5. One-way ANOVA statistical evaluation showed that no substantial difference was observed (*p*>.05) in the assessed parameters between the stored M5 and the original fresh one being compared with it. Similarity factor of *in vitro* CLZ release was calculated and was equal to 95.05 showing that the storage had no obvious effect on drug release. Our findings are in accordance with that declared by Sayed et al. (2017) who observed no alternation in appearance or aggregation of felodipine P123/F127 nanomixed micellar systems after being stored at room temperature and no substantial change was observed upon comparing the computed parameters of fresh and stored formulae, indicating good physical stability of the formed polymeric mixed micelles.

**Table 5.** Effect of storage on the physicochemical properties of the optimized CLZ-loaded PNM formula (M5).

Parameters	Fresh	After 3 months of storage at room temp.
Drug content (%)	101 ± 2.36	96.23 ± 2.05
PS (nm)	217.1 ± 9.32	225 ± 6.65
Q6 h (%)	39.25 ± 4.48	37.25 ± 1.05
Q24 h (%)	92.79 ± 3.20	90.54 ± 5.650

### 3.5. *Ex vivo* characterization of the optimized CLZ-loaded PNMS formula

#### 3.5.1. Histopathological studies

Nasal histopathology was conducted to assess the local toxicity effect of the optimized formula (M5) on the nasal mucosa. The posterior and anterior parts of the nasal sheep mucosa were examined after being treated with M5 in comparison with PBS PH 7.4 (negative control).

**Figure 5.**
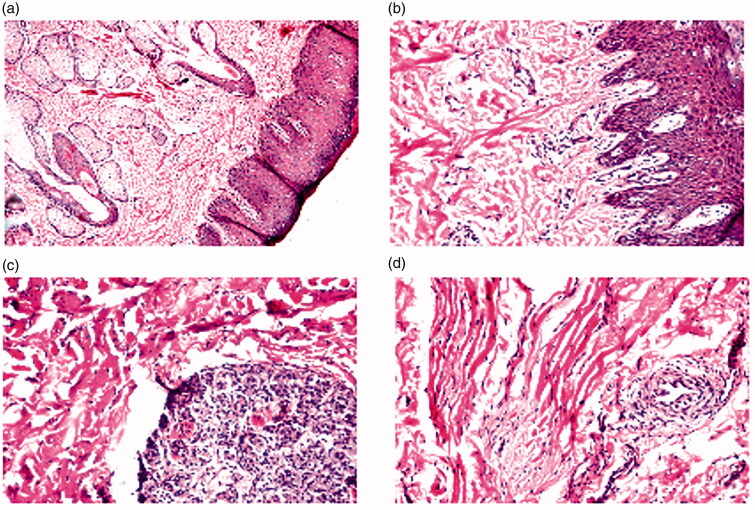
Photomicrographs showing histopathological sections of the *anterior parts* of sheep nasal mucosa treated with PBS pH 7.4 (negative control, a) and M5 (optimized CLZ-loaded PNMS, b). And the *posterior parts* of sheep nasal mucosa treated with PBS pH 7.4 (negative control, c) and M5 (optimized CLZ-loaded PNMS, d).

By examining the anterior part, in normal nasal mucosa treated with PBS (negative control), no histopathological change was found in the usual structure of the lining mucosal stratified squamous keratinized epithelium and the underlying stroma with hair follicles and sebaceous glands as shown in Figure 5(a). Also, upon administration of M5, no substantial alternation in the connective tissues, dermis and epidermis was witnessed compared to the control treated group (Figure 5(b)).

Regarding the posterior part, no histopathological change was present in the nasal mucosa when treated with PBS also, histological structure of the cartilaginous structure, stroma with glands and the musculature was normal (Figure 5(c)). Also, no histopathological alteration in the connective tissues, dermis and epidermis was recorded upon administration of M5 (Figure 5(d)).

Such findings are in agreement with Kolsure & Rajkapoor (2012) who formulated zolmitriptan nanomicelles using two types of copolymers Pluronic F127 and Pluronic^®^ F68, histopathological studies showed that there was no substantial effect in the microscopic mucosal nasal structure as the surface epithelium lining and the granular cellular structure of the nasal mucosa were completely intact.

#### 3.5.2. Permeation through nasal sheep mucosa

**Figure 6.**
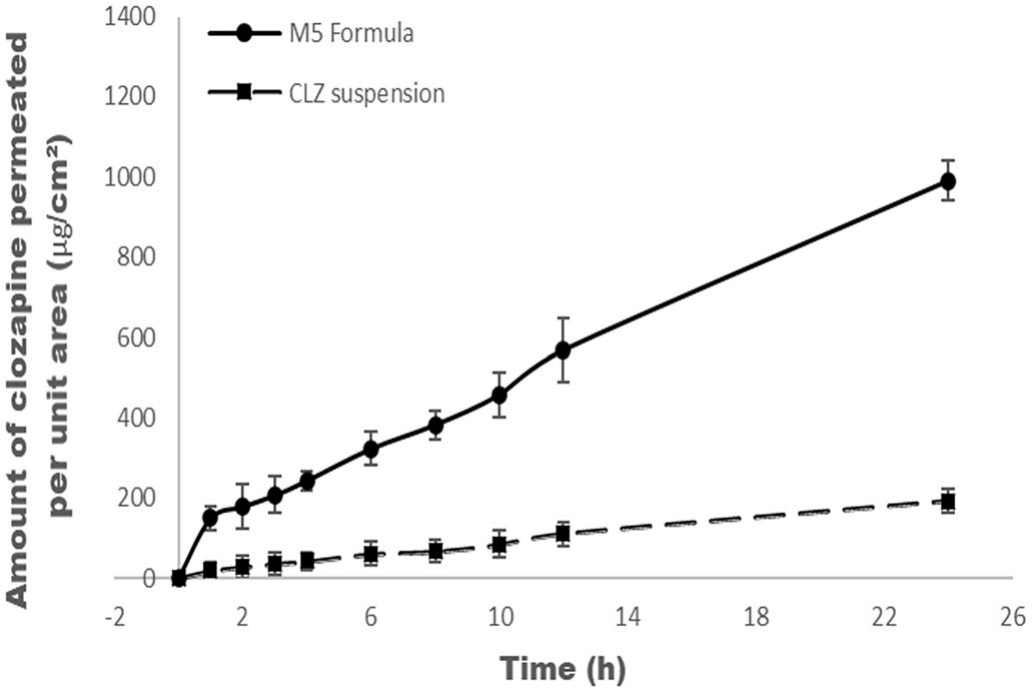
*Ex vivo* permeation profiles of M5 formula and CLZ suspension through nasal sheep mucosa.

*Ex vivo* permeation experiments have been conducted in order to compare the CLZ diffusion from the optimized formula M5 and CLZ suspension (control) using nasal sheep mucosa. Both the optimized formula and the drug suspension had the same CLZ amount (1.4 mg of CLZ/mL). Methanol (40% v/v) was added to the PBS 7.4 receptor compartment to enhance CLZ solubility in addition to maintain sink condition (Shafaat et al., 2013). The amount of CLZ permeated from M5 through nasal sheep mucosa was significantly higher (*p*< .05) than that of CLZ suspension (Figure 6). M5 showed flux equal 36.62 μg/cm^2^.h with enhancement ratio of 5 which means that the flux of M5 is increased by fivefold compared to CLZ suspension flux. The previously mentioned results are compatible with *in vitro* release results which confirm that the nanosized mixed micelles of M5 enhance the amount permeated through the nasal sheep mucosa due to micellar solubilization of CLZ compared to its suspension permeation profile in addition to the inhibition effects of PNMS on P-glycoprotein that present in the blood brain barrier (Salama et al., 2012).

It showed that the mixed micelles increase the resident period between the drug and the nasal sheep mucosa because it protects the drug from the mucociliary clearance due to rapid turnover of the mucus (Ganger & Schindowski, 2018).

### 3.6. *In vivo* characterization of the optimized CLZ-loaded PNMS formula

#### 3.6.1. Radiolabelling of M5

**Figure 7.**
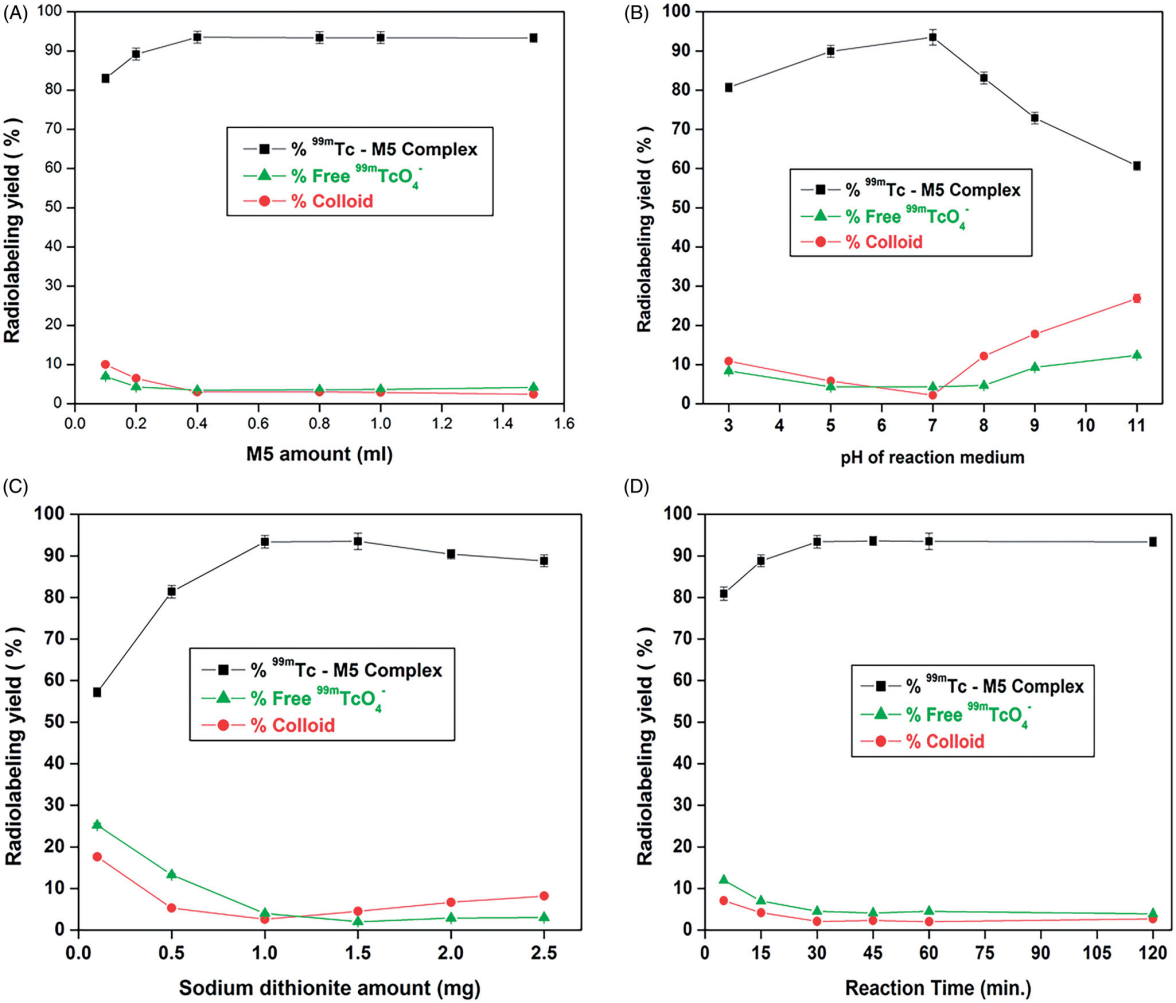
Variation of the radiolabeling yield of ^99m^Tc-M5 complex as a function of M5 amount (A), pH (B), sodium dithionite (Na_2_S_2_O_4_) (C), and reaction time (D).

The optimized formula M5 was efficiently radiolabeled by direct labeling process with ^99m^Tc and the radiolabeling yield was affected by many factors. The reaction conditions variables (amount of both sodium dithionite and M5, pH of medium and reaction time) were optimized to obtain the highest labeling yield as shown in Figure 7(A–D). ^99m^Tc–M5 complex was synthesized in excellent yield (93.5 ± 1.5%) under the optimized conditions by incubating a mixture of 0.4 mL of M5 formula, 1 mg of Na_2_S_2_O_4_, and 0.1 mL of freshly eluted technetium Tc-99m pertechnetate (^99m^TcO_4_^–^, 400 MBq) at room temperature for 30 min and pH of medium was adjusted at 7.

#### 3.6.2. Biodistribution study of ^99m^Tc–M5 complex

**Figure 8.**
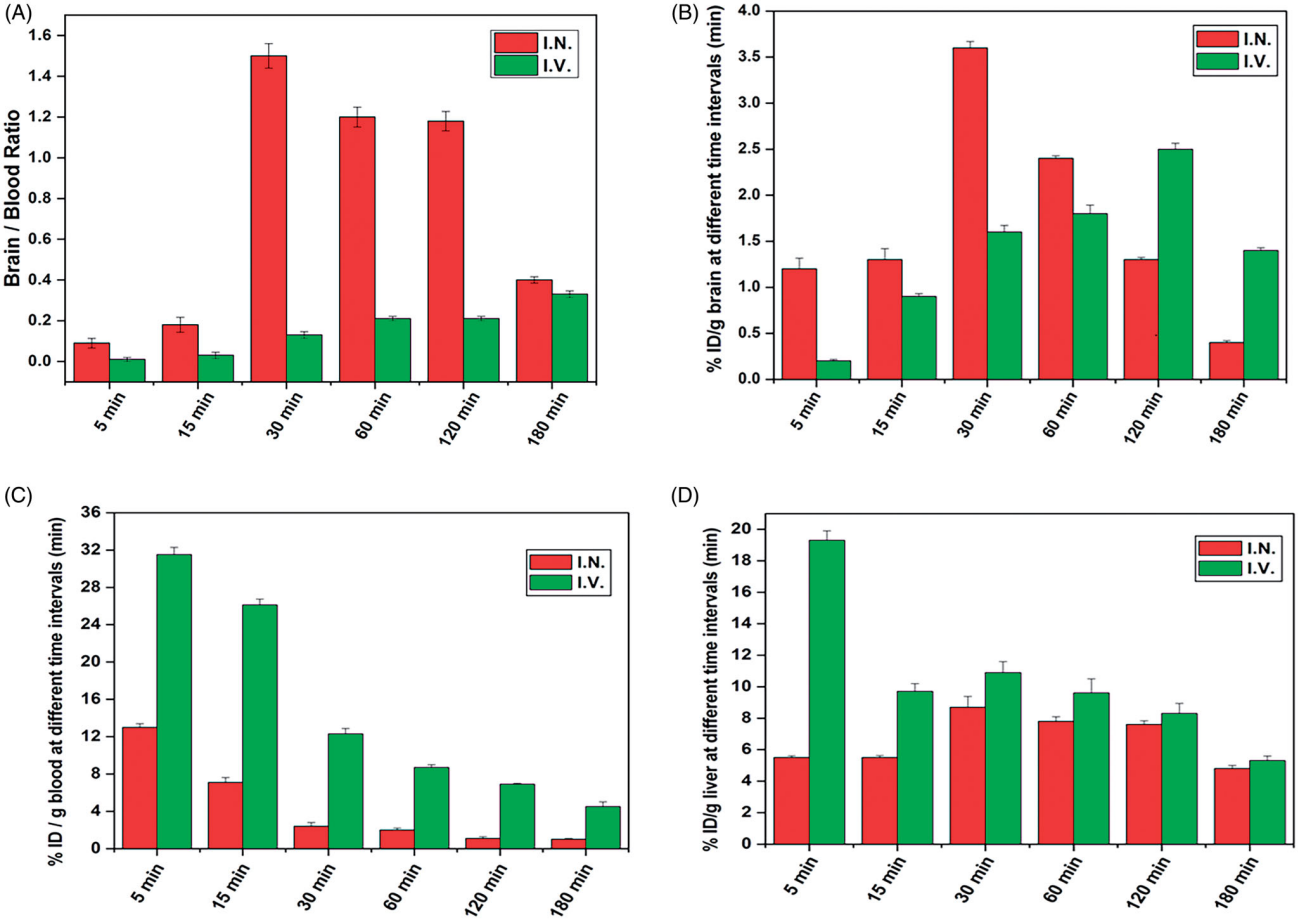
*In vivo* biodistribution comparison of ^99m^Tc–M5 complex at different time intervals post I.N. and I.V. administration where (A) brain-to-blood ratios, (B) brain uptake, (C) blood uptake, and (D) liver uptake.

Comparison between ^99m^Tc–M5 complex IV and IN administration brain-to-blood ratios, brain, blood, and liver uptake is illustrated in Figure 8(A–D). ^99m^Tc–M5 complex has maximum brain/blood ratio (1.5 ± 0.17 at 30 min) following IN administration which stayed upper than 1 up to 120 min then reduced slowly to 0.4 ± 0.03 at 180 min. After IV injections, the brain/blood ratios augmented regularly to plateau with the greatest ratio of 0.33 ± 0.10 was observed at 180 min, which was <1 suggests that the ^99m^Tc–M5 complex diffuses to the blood higher than the brain following IV administration.

The largest brain uptake of ^99m^Tc–M5 complex was 3.6 ± 0.12% ID/g at 30 min and 2.5 ± 0.09% ID/g at 120 min following IN and IV, respectively (Figure 8(B)). A significant higher brain uptake (*p=* .013) following IN administration reveals the power of ^99m^Tc–M5 complex for selective accumulation and localization in brain. The blood uptake of ^99m^Tc–M5 complex after IN administration (2 ± 0.05% ID/g at 60 min) was significantly lower (*p=* .000) than IV administration (8.3 ± 0.15% ID/g at 60 min) which may be useful for tracking systemic radiotracer unwanted effects, like cardiac and extracurricular side effects after IV injection of the ^99m^Tc–M5 complex, results are shown in Figure 8(C).

Intranasal delivery has the advantages over IV delivery regarding brain targeting as IN delivery reach the maximum brain uptake (3.6 ± 0.12% ID/g) in a much quicker time (30 min) with maximum brain/blood ratio (1.5 ± 0.17) (Figure 8(A,B)). Also, the maximum liver uptake of ^99m^Tc–M5 complex was 8.7 ± 0.62% ID/g at 30 min and 19.3 ± 0.34% ID/g at 5 min following IN and IV, respectively, Figure 8(D), approves the biological availability of the ^99m^Tc–M5 complex by bypassing hepatic metabolism, reaching favorite brain uptake of ^99m^Tc–M5 complex.

Pharmacokinetic parameters for IN and IV ^99m^Tc–M5 complex were estimated by calculating the following parameters (*C*_max_, *T*_max_, and AUC (0–180 min)) and represented in Table 6. *C*_max_ of brain after IN administration (3.6 ± 0.42%/g) was significantly greater (*p=* .013) (about 1.5-fold) compared to IV administration of ^99m^Tc–M5 complex (2.5 ± 0.20%/g). In addition, *T*_max_ showed a significant difference (*p=* .000) following IN and IV administration of ^99m^Tc–M5 complex (30 ± 3.73 and 120 ± 10.99 min), respectively. The higher *C*_max_ together with lower *T*_max_ following IN ^99m^Tc–M5 compared to IV administration could be justified by the great ability of PNMS to permeate via the nasal membrane and their inhibition effects of P-glycoprotein in the BBB (Salama et al., 2012). However, AUC_(0–180)_ in brain after IN and IV administration of ^99m^Tc–M5 complex showed a non-significant difference (*p=* .189). It is clear that IN blood *C*_max_ was significantly lower (*p=* .004) than *C*_max_ of the blood after IV administration; 13 ± 5.07 and 31.5 ± 2.05%ID/g, respectively. This indicates that IN has advantage over IV by having lower side effects and by passing the hepatic first pass metabolism. The drug targeting efficiency (DTE) was found to be 396.5% confirming that the optimized CLZ-loaded PNMS prospered to diffuse via the nasal mucosa and transfer fourfold superior amount of CLZ at the chief target place (Jönsson et al., 2019) following IN administration compared to IV administration.

**Table 6.** The mean pharmacokinetic parameters of M5 in blood and brain of mice after administration of single dose of M5 via the IV and IN route.

	IN M5		IV M5	
	Blood	Brain	Blood	Brain
*C*_max_ (%ID/g)	13 ± 5.07	3.6 ± 0.42	31.5 ± 2.05	2.5 ± 0.20
*T*_max_ (min)	5 ± 0.25	30 ± 3.73	5 ± 0.12	120 ± 10.99
AUC_(0–180)_ (min %ID/g)	426.25 ± 22.12	304.25 ± 11.09	1780 ± 82.29	321.75 ± 15.62
MRT (min)	51.99 ± 4.15	68.10 ± 25	62.84 ± 2.34	88.45 ± 1.15

Built on the research done by Serralheiro et al. (2014), the boosted brain uptake of ^99m^Tc–M5 complex following IN administration can be justified in terms of the relationship of direct and indirect uptake routes. After IN administration, a fraction enters the brain directly via the olfactory and/or the trigeminal nerves. The direct route provides the major fraction of ^99m^Tc–M5 complex. However, additional fraction reached the systemic circulation by transcellular transportation via the nasal membrane and then passes the BBB reaching the brain. The indirect route supplies the brain with small fraction of ^99m^Tc–M5 complex.

## 4. Conclusions

In this study, CLZ was successfully formulated and optimized as stable IN PNMS with good nasal tolerability. PNMS had the ability of increase CLZ solubility up to 78 times (14.73 mg/mL) compared to its aqueous solubility (0.1889 mg/mL) also showing a good permeation behavior with fivefold higher flux when compared to CLZ suspension. It has been elucidated that biodistribution and pharmacokinetics studies following radiolabeling of the optimum CLZ-loaded formula (^99m^Tc–M5) when administrated IN showed effective brain uptake (3.6 ± 0.12% ID/g) with higher brain/blood ratio (1.5 ± 0.17) at 30 min and superior DTE compared to IV administration (2.5 ± 0.09% ID/g at 120 min) and (0.33 ± 0.1 observed at 180 min), respectively. Consequently, IN application of CLZ-loaded PNMS could be an encouraging brain targeting delivery system for successful treatment of schizophrenia.
